# Sex and Ethnic Differences in 47 Candidate Proteomic Markers of Cardiovascular Disease: The Mayo Clinic Proteomic Markers of Arteriosclerosis Study

**DOI:** 10.1371/journal.pone.0009065

**Published:** 2010-02-05

**Authors:** Charles X. Kim, Kent R. Bailey, George G. Klee, Allison A. Ellington, Guanghui Liu, Thomas H. Mosley, Hamid Rehman, Iftikhar J. Kullo

**Affiliations:** Mayo Clinic, Rochester, Minnesota, United States of America; University of Giessen Lung Center, Germany

## Abstract

**Background:**

Cardiovascular disease (CVD) susceptibility differs between men and women and varies with ethnicity. This variability is not entirely explained by conventional CVD risk factors. We examined differences in circulating levels of 47 novel protein markers of CVD in 2561 men and women of African-American (AA) and non-Hispanic White (NHW) ethnicity, enrolled at geographically distinct sites.

**Methodology/Principal Findings:**

Participants (1,324 AAs, mean age 63.5 y, 71% women; 1,237 NHWs, mean age 58.9 y, 57% women) belonged to sibships ascertained on the basis of hypertension. Solid-phase immunoassays and immunoturbidometric, clot-based, chromogenic, and electrophoretic assays were used to measure the 47 protein markers in plasma or serum. Marker levels were log transformed and outliers were adjusted to within 4 SD. To identify markers independently associated with sex or ethnicity, we employed multivariable regression analyses that adjusted for conventional risk factors, prior history of CVD, medication use and lifestyle factors (physical activity, alcohol consumption and education). Generalized estimating equations were used to correct for intrafamilial correlations. After adjustment for the above covariates, female sex was associated with higher levels of 29 markers and lower levels of 6 markers. Female sex was independently associated with higher levels of several inflammatory markers as well as lipoproteins, adipokines, natriuretic peptides, vasoconstrictor peptides and markers of calcification and thrombosis. AA ethnicity was associated with higher levels of 19 markers and lower levels of 6 markers, including higher levels of several inflammatory makers, higher leptin and lower adiponectin levels, lower levels of vasodilator-natriuretic peptides, higher levels of vasoconstrictor-antidiuretic peptides and markers of calcification and thrombosis.

**Conclusions/Significance:**

Plasma levels of several novel protein markers of CVD differ significantly in the context of sex and ethnicity. These results have implications for individualized CVD risk assessment.

## Introduction

Algorithms based on several established risk factors are used in the clinical setting for stratifying the risk of cardiovascular disease (CVD) in asymptomatic individuals [1], [2], [3], [4]. However, these risk-stratification algorithms are limited in their ability to discriminate which individuals will suffer adverse cardiovascular events [5], [6]. Several methods have been proposed to improve specificity of cardiovascular risk stratification [7], [8], [9]. Advances in our knowledge of the pathophysiology of arteriosclerotic vascular disease have highlighted its complex etiology and led to the proposal of a “multimarker approach” for risk stratification [9]. Although novel biomarkers hold promise for refining CVD risk stratification and formulating tailored strategies to improve quality-of-life and reduce mortality [10], [11], [12], reliable and reproducible assays of circulating protein markers are often unavailable [13]. Furthermore, the effects of sex and ethnicity on the plasma levels of key candidate protein markers have not been fully described.

The Mayo Vascular Proteomics Program was funded by the National Heart, Lung and Blood Institute to investigate multimarker approaches for early detection of CVD. Protein markers (n = 47, see Table 1 for abbreviations) were selected from pathways of inflammation, lipoprotein metabolism, adipocyte metabolism, hemodynamic stress, calcification and thrombosis. Markers were selected based on basic science, observational and clinical studies suggesting the roles of these markers in arteriosclerosis and in mediating end-organ damage in the context of hypertension. Uniplex and multiplex assays were used to measure the 47 markers in stored blood samples of 1324 African-American (AA) and 1237 non-Hispanic white (NHW) participants. In this report, we describe the associations of sex and ethnicity with circulating levels of these 47 protein markers.

**Table 1:** Protein marker abbreviations.

CRP, C-reactive protein	SAA, serum amyloid A	ICAM, Intercellular adhesion molecule	VCAM, vascular cell adhesion molecule	IL-6, interleukin 6
IL-18, interleukin 18	TNFRI, tumor necrosis factor receptor-1	TNFRII, tumor necrosis factor receptor-2	MCP-1, monocyte chemotactic protein-1	Hsp27, heat shock protein 27
MPO, myeloperoxidase	RAGE, receptor for advanced glycation endproducts	MMP-2, matrix metalloproteinase-2	MMP-9, matrix metalloproteinase-9	TIMP-1, tissue inhibitor of metalloproteinases-1
TIMP-2, tissue inhibitor of metalloproteinases-2	ApoA-I, apolipoprotein A-I	ApoB, apolipoprotein B	ApoC-III, apolipoprotein C-III	ApoE, apolipoprotein E
LDL, low-density lipoprotein	Lp(a), lipoprotein (a)	Ox-LDL, oxidized low-density lipoprotein	Lp-PLA_2_, lipoprotein-associated phospholipase A2	NT-proBNP, N-terminal pro-brain natriuretic peptide
MR-proANP, midregional pro-atrial natriuretic peptide	CT-proAVP, C-terminal pro-arginine vasopressin	MR-proADM, midregional pro-adrenomedullin	CT-proET, C-terminal pro-endothelin	OPN, osteopontin
OPG, osteoprotegerin	ONN, osteonectin	OCN, osteocalcin	vWF, von Willebrand Factor	ATIII, antithrombin III

## Methods

### Study Population

Subjects included participants in the Genetic Epidemiology Network of Arteriopathy (GENOA) study, a multicenter community-based study to identify genes influencing blood pressure (BP) and development of target organ damage due to hypertension [11], [14]. These cohorts are enriched for hypertension and thereby suitable for identifying markers associated with subclinical vascular disease. The AA participants were recruited from Jackson in Hinds County, Mississippi, while the NHW participants were recruited from Rochester in Olmsted County, MN. The Jackson, MS cohort of the Atherosclerosis Risk in Communities study [15], originally a probability sample of persons with driver's licenses, was used to ascertain AA sibships. The sampling frame of the Rochester GENOA cohort was the Mayo Clinic diagnostic index and medical record linkage system of the Rochester Epidemiology Project [16]. It was used to identify NHW residents of Olmsted County, MN, diagnosed with essential hypertension before age 60. If the eligible proband had at least one sibling with hypertension, all available full biologic siblings of the index hypertensive, including normotensive siblings, were invited to participate in interviews, physical examinations, and phlebotomy at their respective centers. The only exclusionary criterion at enrollment at either center was the presence of a secondary cause of hypertension (such as documented renal artery stenosis or advanced renal insufficiency) in the index sibs.

In Phase I of the study, sibships with at least 2 individuals diagnosed with essential hypertension prior to the age of 60 years were enrolled in Jackson, MS (AA subjects, *n* = 1854) and Rochester, MN (NHW subjects, *n* = 1583). Between December 1, 2000, and October 31, 2004, the Phase I GENOA participants returned for a second study visit and underwent physical examination, provided blood samples and underwent characterization of subclinical markers of arteriosclerosis. Blood was collected by venipuncture after an overnight fast and processed using standardized protocols at each collection site. Blood was centrifuged for 10 min at 4°C, aliquoted in 0.5–1 mL volumes of sodium-citrate plasma, EDTA plasma, and serum and stored at −80°C within 2 h of venipuncture. Aliquots of AA samples frozen to −80°C were shipped to Rochester, MN overnight on dry ice. Samples were visually inspected for evidence of thawing and then stored at −80°C. For protein measurements, samples were thawed on ice and aliquoted into bar-coded Eppendorf tubes. The new sample aliquots were re-frozen to −80°C until time of testing at which point they were thawed on ice again. Thus, samples from each collection site were exposed to identical numbers of freeze-thaw cycles for a given assay, ensuring that differential sample handling would not contribute to any subsequently noted ethnic differences in protein levels. The study was approved by the Institutional Review Boards of the University of Mississippi Medical Center, Jackson, MS, and Mayo Clinic, Rochester, MN, and participants gave informed consent.

### Conventional Risk Factors

Standardized protocols were used by trained study coordinators in all examinations. Height was measured by stadiometer and weight by electronic balance to calculate body mass index (BMI) (kg/m^2^). Resting systolic and diastolic BP levels were measured with a random zero sphygmomanometer in the right arm. The diagnosis of hypertension was based on either BP measurements (>140/90 mm Hg) or previous diagnosis of hypertension and current treatment with anti-hypertensive medications. Diabetes was considered present if a participant was receiving treatment with insulin, oral agents, or had fasting serum glucose levels ≥126 mg/dL. Information about the use of BP medications, statins, and estrogen use was obtained from questionnaires completed by the participants. Serum cholesterol, high-density lipoprotein (HDL) cholesterol, glucose, and creatinine were measured by standard enzymatic methods.

### Lifestyle Variables

Information on three ‘lifestyle’ variables – physical activity, alcohol consumption, and education – was obtained from a questionnaire administered to the participants. We constructed a physical activity scale using responses to questions on how many hours per day of heavy activity, moderate activity, slight activity, and sedentary activity the participant engaged in. Specifically, the physical activity score was derived as follows: 2*heavy + moderate – sedentary (hours). Alcohol intake was quantified as ounces of alcohol per month and was estimated from the type and frequency of beverage consumed. Education was recorded as years in school.

### Measurement of Candidate Biomarkers

The 47 candidate protein markers of vascular disease were measured in plasma (EDTA or citrate) or serum using solid-phase immunoassays (n = 32) and immunoturbidometric (n = 7), clot-based (n = 5), chromogenic (n = 2), and electrophoretic (n = 1) assays using commercially available reagents (Table S1). MR-proADM, CT-proAVP, CT-proET, and MR-proANP were measured as part of a research collaboration with BRAHMS AG (Henningsdorf, Germany). Given the logistical difficulties of measuring a large number of markers in 2561 participants, a subset of markers were measured using multiplex assays; IL-6, IL-18, P-selectin, RAGE, TNFRI, E-selectin, MCP-1, MMP-2, MMP-9, TIMP-1, TIMP-2, TNFRII, and ICAM were measured using a contracted service with SearchLight™ Technologies (Boston, MA). The markers of calcification (OPN, OPG, ONN, OCN) were measured in the investigator's laboratory using the Meso Scale Discovery immunoassay platform (Gaithersburg, MD). The remaining proteins were either measured in investigator's laboratory or the Mayo Immunochemical Core Laboratory (Rochester, MN).

### Technical Assay Performance

We evaluated intra- and inter-assay imprecision at a minimum of one level for each analyte to assess technical assay performance (please see supplementary Table S1). Precision data for MR-proADM[17], CT-proAVP[18], CT-proET[19], and MR-proANP[20] were derived from previous reports. For analytes measured at Mayo, we prospectively determined intra-assay imprecision (reported as coefficient of variation (CV)) by measuring the candidate protein markers in blood samples from volunteers in 10 parallel measurements, and inter-assay imprecision (CV) by measuring the same samples across 10 assay runs. We retrospectively determined precision for the assays performed by SearchLight™ based on data derived from a blinded, internal plasma control sample. Due to plate-to-plate variations in protein levels in the SearchLight data sets, we developed an algorithm to reduce inter-plate variability; normalized data were used for subsequent analyses [13].

### Quality Control

Our quality control program included evaluation of intra-assay imprecision between duplicate sample measurements and inter-assay imprecision of quality control materials. We measured protein levels in duplicate, except for Lp-PLA_2_ mass and activity, for which only single measurements were made. Sample measurements with CVs >20% were either retested or excluded from the dataset. Acceptable imprecision of measurements from the SearchLight™ platform was set at <30% due to performance limitations; mean values of samples with CVs >30% were replaced with the singlet value closest to the plate median because retesting was not feasible. We monitored inter-assay imprecision by measuring 1–3 quality control materials as part of each assay run, and we interpreted the results using a multi-rule approach (1_3s_ and 2_2s_ Westgard rules) [21]. These rules reject all data included in an assay run if any level of QC material was three standard deviations (SD) above or below the target value or if 2 or more levels were 2 SD beyond the target value in the same direction. Only the 1_3s_ rule was applied to the OPN assay due to a problem with the second level of QC material. With the exception of proteins measured on the SearchLight™ platform, acceptable coefficient of variance between inter-plate measurements was <20% for all assays and analyses were performed in real-time. Two levels of SearchLight™ controls and one normal serum control were embedded randomly across study plates and evaluated retrospectively using a modified multi-rule approach as described elsewhere [13]. Sample measurements from failed plates were either repeated or excluded from the data set.

### Statistical Analysis

Circulating levels of markers were log transformed due to skewed distribution and data beyond 4 SD were identified as outliers and winsorized to the minimal or maximal value within 4 SD. Multiple imputation (stratified by ethnic group) using PROC MI in SAS was applied to impute missing values for the protein markers (range 0-29.9%; SAA 17.5%, ICAM 21.8%, IL-18 16.0%, TNFRI 16.9%, MCP-1 15.9%, RAGE 15.4%, NT-proBNP 20.2%, MMP-9 29.9%, TIMP-2 17.2%, OPN 26.9%). All analyses were performed in SAS 9.1.3 (SAS Institute Inc., Cary, NC).

Age- and BMI-adjusted geometric means of the 47 protein markers were compared in men and women of the two ethnic groups using the Wald chi-square test. We investigated whether sex was independently associated with circulating levels of biomarkers using multivariable regression analysis, after adjusting for conventional cardiovascular risk factors (age, smoking, hypertension, total cholesterol, HDL cholesterol, diabetes), history of CVD, a measure of adiposity (BMI), medication use (antihypertensives, statins, aspirin, and in women, estrogen), lifestyle variables, and estimated glomerular filtration rate (eGFR). Adjustment for total and HDL cholesterol was not performed for the lipoproteins ApoA-I, ApoB, ApoC-III, and ApoE. Similar multivariate regression analyses, stratified by sex, were used to identify markers independently associated with ethnicity. Because of sibships in the sample, population-averaged generalized estimating equations[22] were used to account for the impact of familial correlations on the relationships between independent and dependent variables.

## Results

Clinical characteristics of the study population are listed in Table 2. AA were older, had higher BP levels, were more often diabetic, had lower physical activity scores, and lower statin and aspirin use. There were higher rates of smoking in AA men and NHW women. The age and BMI-adjusted geometric means of the protein markers in men and women, stratified by ethnicity, are shown in Table 3.

**Table 2:** Participant characteristics and ethnic differences.

		Women (n = 1638)			Men (n = 923)		
	N	AA (n = 936)	NHW (n = 702)	*P*	AA (n = 388)	NHW (n = 535)	*P*
Age, years	2561	63.3±9.4	58.4±10.3	<.0001	64.3±9.0	59.5±10.0	<.0001
BMI, kg/m^2^	2557	32.5±7.0	30.8±7.1	<.0001	29.2±4.9	30.7±5.1	<.0001
Total cholesterol, mg/dL	2561	205.9±41.0	202.8±34.9	0.1078	191.9±41.0	190.0±32.7	0.4335
HDL cholesterol, mg/dL	2561	61.0±18.1	57.4±15.4	<.0001	49.3±15.6	44.5±11.4	<.0001
Systolic BP, mm Hg	2559	139.5±21.2	131.7±17.9	<.0001	136.3±20.1	130.3±15.9	<.0001
Diastolic BP, mm Hg	2559	78.2±10.6	72.8±9.2	<.0001	81.1±10.9	75.3±9.1	<.0001
Ever smoker, %	2651	289 (30.9)	284 (40.5)	0.0001	250 (64.4)	326 (60.9)	0.2787
Diabetes, %	2651	282 (30.1)	92 (13.1)	<.0001	109 (28.1)	93 (17.4)	0.0001
Previous history of MI or stroke	2651	78 (8.3)	37 (5.3)	0.0164	53 (13.7)	55 (10.3)	0.1149
Statin use, %	2651	170 (18.2)	165 (23.5)	0.0080	76 (19.6)	197 (36.8)	<.0001
Aspirin use, %	2651	287 (30.7)	243 (34.6)	0.0906	149 (38.4)	267 (49.9)	0.0005
Physical activity score	2561	9.6±3.1	12.8±4.9	<.0001	10.2±4.1	14.1±5.6	<.0001
Alcohol (oz)/month	2548	0.8±3.9	3.1±6.0	<.0001	3.2±7.9	9.1±14.1	<.0001
Education, years	2561	12.3±3.4	13.4±2.2	<.0001	11.6±4.2	13.5±2.6	<.0001
eGFR, mg/dL	2561	75.3±19.8	63.7±13.6	<.0001	74.5±19.9	65.4±13.7	<.0001

AA, African American; NHW, non-Hispanic white; BMI, body mass index; HDL, high-density lipoprotein; BP, blood pressure; eGFR, estimated glomerular filtration rate; MI, myocardial infarction.

**Table 3:** Sex and ethnic differences in circulating levels of 47 protein markers (adjusted for age and BMI).

		Women (n = 1638)			Men (n = 923)		
	N	AA (n = 936)	NHW (n = 702)	*P*	AA (n = 388)	NHW (n = 535)	*P*
**Inflammation**							
CRP, mg/L	2550	3.59***	3.33***	0.1528	2.91	2.10	<.0001
SAA, µg/mL	2113	22.63***	22.77***	0.9013	16.91	15.94	0.3587
ICAM, ng/mL	2003	274.48*	284.74	0.0334	260.41	279.91	0.0015
VCAM, ng/mL	2496	570.21	687.62	<.0001	558.63	670.71	<.0001
IL-6, pg/mL	2220	7.98	7.30	0.0053	7.90	7.78	0.7422
IL-18, pg/mL	2151	60.49	69.58**	<.0001	61.58	75.70	<.0001
TNFRI, pg/mL	2129	1027.66	1266.95	<.0001	1046.58	1302.81	<.0001
TNFRII, pg/mL	2222	1731.16	1857.16	0.0012	1751.27	1889.10	0.0029
MCP-1, pg/mL	2153	978.56	859.33*	<.0001	980.77	901.42	0.0012
E-selectin, ng/mL	2225	68.85	69.36*	0.6689	70.69	72.19	0.3334
P-selectin, ng/mL	2186	30.09**	27.66***	0.0016	32.80	32.87	0.9512
Hsp27, ng/mL	2206	1726.27	1484.84**	0.0041	1721.46	1274.73	<.0001
MPO, ng/mL	2379	36.56	28.54**	<.0001	34.70	25.08	<.0001
RAGE, pg/mL	2167	437.96***	597.53	<.0001	359.32	560.34	<.0001
MMP-2, ng/mL	2221	1807.08	1693.05	0.0015	1808.72	1705.78	0.0142
MMP-9, ng/mL	1794	29.18	34.66	<.0001	30.01	36.16	<.0001
TIMP-1, ng/mL	2219	68.47***	83.92***	<.0001	75.18	92.97	<.0001
TIMP-2, ng/mL	2120	156.29	148.24	0.0013	152.29	146.87	0.0556
**Lipoprotein metabolism**							
ApoA-I, mg/dL	2391	164.85***	169.53***	0.0358	137.75	139.30	0.5150
ApoB, mg/dL	2330	92.22	97.61	0.0001	89.77	97.96	<.0001
ApoC-III, mg/dL	2261	14.02***	18.19***	<.0001	12.98	16.32	<.0001
ApoE, mg/dL	2260	5.26***	5.14***	0.2681	4.68	4.79	0.3739
LDL size, nm	2561	269.34***	270.60***	<.0001	267.89	269.28	<.0001
Lp(a), mg/dL	2561	46.83***	15.76*	<.0001	35.80	13.85	<.0001
Ox-LDL, U/L	2275	62.31	65.69	0.0024	63.48	65.94	0.0882
Lp-PLA_2_ mass, ng/mL	2294	204.34*	242.36**	<.0001	211.39	253.29	<.0001
Lp-PLA_2_ activity, mol/min/mL	2227	119.30***	129.67***	<.0001	137.11	154.89	<.0001
**Adipocyte metabolism**							
Leptin, ng/mL	2288	26.51***	22.46***	<.0001	9.62	8.81	0.0337
Adiponectin, µg/mL	2424	10.11***	13.07***	<.0001	7.65	9.56	<.0001
Resistin, ng/mL	2308	3.62**	3.76***	0.1519	3.24	3.39	0.1975
**Hemodynamics**							
NT-proBNP, pg/mL	2043	79.74	127.57***	<.0001	84.77	107.28	<.0001
MR-proANP, pmol/L	2548	60.53*	71.16***	<.0001	55.82	58.60	0.2267
CT-proAVP, pmol/L	2511	7.25***	4.72***	<.0001	9.54	6.55	<.0001
MR-proADM, nmol/L	2501	0.53***	0.60***	<.0001	0.49	0.54	<.0001
CT-proET, pmol/L	2366	42.15*	40.31	0.0892	44.91	42.24	0.0297
**Calcification**							
OPN, pg/mL	1872	25.61*	18.81***	<.0001	28.04	23.37	0.0002
OPG, pg/mL	2297	307.44***	265.88***	<.0001	284.20	238.56	<.0001
ONN, pg/mL	2232	945.24	895.78	0.0041	925.35	915.86	0.6769
OCN, pg/mL	2193	14.57*	11.32***	<.0001	13.57	12.90	0.1351
**Thrombosis**							
Factor II, % activity	2341	110.67***	105.01***	<.0001	103.12	98.20	0.0022
Factor V, % activity	2405	102.40	93.77	<.0001	100.00	93.03	<.0001
Factor VII, % activity	2424	114.65***	122.45***	0.0001	100.84	105.50	0.0462
Factor VIII, % activity	2363	148.48**	128.95*	<.0001	139.13	122.43	<.0001
vWF, IU/dL	2468	178.52*	147.92	<.0001	169.99	145.41	<.0001
D-dimer, ng/mL	2397	208.07*	181.12***	0.0001	188.41	152.58	<.0001
ATIII, % activity	2445	108.81***	106.83	0.0056	105.62	105.53	0.9225
Fibrinogen, mg/dL	2552	363.16***	313.93	<.0001	342.38	313.30	<.0001

AA, African American; NHW, non-Hispanic white; BMI, body mass index.

Geometric means were presented after adjusted by age and BMI, based on the log transformed value after winsorization and imputation.

*, **, *** indicate the significance levels for sex difference in each ethnic group are 0.05, 0.01, and 0.001, respectively.

**Figure 1:**
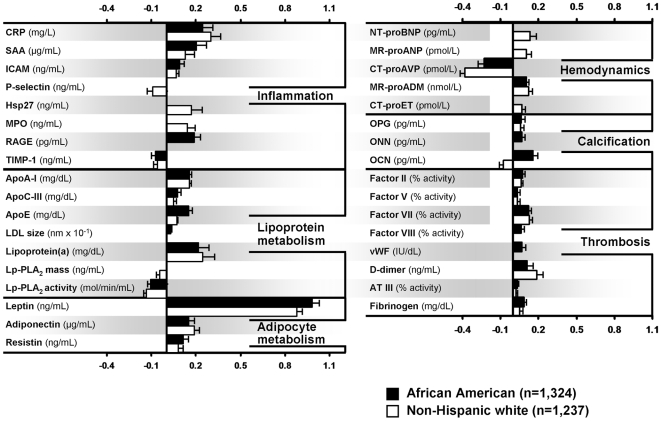
Association of female sex with circulating levels of protein markers. Multivariable regression analyses; markers independently associated with female sex (graphical representation of Table 4, *P*<0.05, β±SE for 1 log change in a marker level is shown).

After adjustment for age, BMI, conventional risk factors, prior history of CVD, medication use, and lifestyle factors, female sex was associated with higher levels of 29 markers and lower levels of 6 markers (Table 4, Figure 1). Differences were largely consistent across the two ethnic groups and spanned all pathways studied. Female sex was associated with higher levels of several inflammatory biomarkers (CRP, SAA, ICAM, Hsp27, MPO and RAGE), apolipoproteins (ApoA-I_,_ ApoC-III_,_ ApoE, Lp(a)), larger LDL particle size, higher levels of adipokines (leptin, adiponectin and resistin), vasodilator peptides (NT-proBNP in NHW, MR-proANP and MR-proADM, vasoconstrictor peptide CT-proET in NHW, calcification markers (OPG, ONN, and OCN (in AA)) and thrombotic markers (Factors II, V, VII, and VIII, vWF, D-dimer, ATIII and fibrinogen). Female sex was associated with lower levels of inflammatory markers P-selectin and TIMP-1, Lp-PLA_2_ mass and activity, vasoconstrictor peptide CT-proAVP and calcification marker OCN. The strongest positive associations were for leptin (β±SE = 0.99±0.04 in AA) and CRP (β±SE = 0.30±0.06 in NHW) and the strongest inverse associations were with CT-proAVP (β±SE = -0.38±0.05 in NHW) and Lp-PLA_2_ activity (β±SE = -0.13±0.02 in NHW).

**Table 4:** Markers independently associated with female sex; multivariable regression analyses.

	African American, n = 1324		non-Hispanic White, n = 1237	
Protein marker	β±SE	*P*	β±SE	*P*
CRP, mg/L	0.243±0.070	0.0005	0.302±0.063	<.0001
SAA, µg/mL	0.205±0.067	0.0022	0.130±0.061	0.0328
ICAM, ng/mL	0.091±0.026	0.0005	0.066±0.022	0.0022
P-selectin, ng/mL	−0.021±0.034	0.5288	−0.093±0.038	0.0144
Hsp27, ng/mL	0.029±0.070	0.6758	0.170±0.076	0.0252
MPO, ng/mL	0.060±0.040	0.1327	0.140±0.053	0.0079
RAGE, pg/mL	0.191±0.038	<.0001	0.080±0.043	0.0625
TIMP-1, ng/mL	−0.074±0.025	0.0026	−0.060±0.025	0.0164
ApoA-I, mg/dL	0.158±0.014	<.0001	0.154±0.016	<.0001
ApoC-III, mg/dL	0.076±0.025	0.0028	0.048±0.023	0.0387
ApoE, mg/dL	0.150±0.026	<.0001	0.072±0.023	0.0016
LDL size, nm	0.003±0.001	0.0079	0.001±0.001	0.3312
Lp(a), mg/dL	0.219±0.068	0.0013	0.246±0.080	0.0022
Lp-PLA_2_ mass, ng/mL	−0.007±0.019	0.7294	−0.045±0.021	0.0323
Lp-PLA_2_ activity, mol/min/mL	−0.107±0.019	<.0001	−0.132±0.020	<.0001
Leptin, ng/mL	0.985±0.043	<.0001	0.880±0.037	<.0001
Adiponectin, µg/mL	0.151±0.039	0.0001	0.188±0.032	<.0001
Resistin, ng/mL	0.113±0.038	0.0025	0.083±0.032	0.0088
NT-proBNP, pg/mL	−0.088±0.050	0.0769	0.136±0.044	0.0021
MR-proANP, pmol/L	0.023±0.038	0.5414	0.101±0.037	0.0062
CT-proAVP, pmol/L	−0.233±0.044	<.0001	−0.381±0.048	<.0001
MR-proADM, nmol/L	0.101±0.019	<.0001	0.124±0.026	<.0001
CT-proET, pmol/L	−0.001±0.026	0.9723	0.066±0.029	0.0252
OPG, pg/mL	0.061±0.025	0.0146	0.059±0.024	0.0156
OPN, pg/mL	0.070±0.023	0.0023	−0.007±0.026	0.7803
OCN, pg/mL	0.155±0.037	<.0001	−0.076±0.034	0.0264
Factor II, % activity	0.073±0.017	<.0001	0.061±0.015	0.0001
Factor V, % activity	0.034±0.016	0.0332	0.035±0.015	0.0247
Factor VII, % activity	0.123±0.023	<.0001	0.129±0.021	<.0001
Factor VIII, % activity	0.064±0.024	0.0081	0.024±0.027	0.3690
vWF, IU/dL	0.070±0.028	0.0127	−0.002±0.031	0.9363
D-dimer, ng/mL	0.112±0.045	0.0124	0.187±0.046	<.0001
ATIII, % activity	0.033±0.008	0.0001	0.024±0.009	0.0059
Fibrinogen, mg/dL	0.087±0.016	<.0001	0.053±0.019	0.0043

Protein marker levels were natural log-transformed and adjusted for age, BMI, smoking, hypertension, diabetes, myocardial infarction, stroke, total and HDL cholesterol, eGFR, systolic blood pressure, medication use (aspirin, estrogen, statin), alcohol, physical activity, education; no total and HDL cholesterol adjustment was done for ApoA-I, ApoB, ApoC-III, and ApoE.

**Figure 2:**
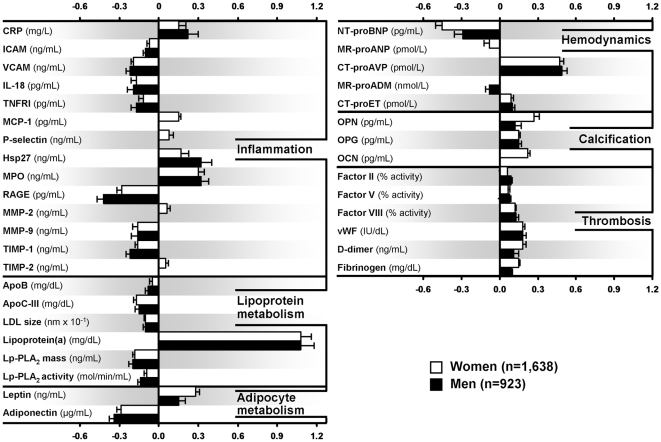
Association of African American ethnicity with circulating levels of protein markers. Multivariable regression analyses; markers independently associated with African American ethnicity (graphical representation of Table 5, *P*<0.0, β±SE for 1 log change in a marker level is shown).

AA ethnicity was associated with higher levels of 19 markers and lower levels of 6 markers after adjustment for age, BMI, conventional risk factors, prior history of CVD, medication use, and lifestyle factors (Table 5, Figure 2). Differences were largely consistent in both men and women, and spanned all studied pathways. AA ethnicity was associated with higher levels of inflammatory biomarkers (CRP, MCP-1, P-selectin, Hsp27, MPO, MMP-2 and TIMP-2), Lp(a), leptin and vasoconstrictor peptides (CT-proAVP, CT-proET), markers of calcification (OPG and OCN), and thrombosis (Factors II, V, and VIII, vWF, D-dimer, fibrinogen). AA ethnicity was associated with lower levels of several inflammatory markers (ICAM, VCAM, IL-18, TNFRI, RAGE, MMP-9, TIMP-1), lipoproteins (ApoB, ApoC-III, LDL size, Lp-PLA_2_ mass and activity), adiponectin and vasodilator peptides (NT-proBNP, MR-proANP, MR-proADM). The strongest positive association was for Lp(a) (β±SE = 1.08±0.10 in men), although MPO and CT-proAVP both had strong associations as well (β±SE = 0.32±0.06 and 0.49±0.05, respectively in men). The strongest inverse associations with AA ethnicity were RAGE (β±SE = −0.42±0.05 in men), adiponectin (β±SE = −0.34±0.04 in men), and NT-proBNP (β±SE = −0.45±0.04 in women).

**Table 5:** Markers independently associated with African American ethnicity; multivariable regression analyses.

	Women, n = 1638		Men, n = 923	
Protein marker	β±SE	*P*	β±SE	*P*
CRP, mg/L	0.15±0.06	0.0167	0.22±0.08	0.0048
ICAM, ng/mL	−0.07±0.02	0.0011	−0.10±0.02	<.0001
VCAM, ng/mL	−0.19±0.02	<.0001	−0.22±0.03	<.0001
IL-18, pg/mL	−0.17±0.04	<.0001	−0.19±0.05	0.0001
TNFRI, pg/mL	−0.12±0.03	0.0003	−0.17±0.04	<.0001
MCP-1, pg/mL	0.15±0.02	<.0001	0.06±0.03	0.0574
P-selectin, ng/mL	0.08±0.03	0.0068	−0.01±0.04	0.8340
Hsp27, ng/mL	0.17±0.06	0.0056	0.32±0.08	0.0001
MPO, ng/mL	0.30±0.05	<.0001	0.32±0.06	<.0001
RAGE, pg/mL	−0.28±0.04	<.0001	−0.42±0.05	<.0001
MMP-2, ng/mL	0.06±0.03	0.0134	0.03±0.03	0.3286
MMP-9, ng/mL	−0.16±0.04	<.0001	−0.16±0.05	0.0005
TIMP-1, ng/mL	−0.16±0.02	<.0001	−0.22±0.03	<.0001
TIMP-2, ng/mL	0.05±0.02	0.0155	0.01±0.02	0.7831
ApoB, mg/dL	−0.05±0.02	0.0024	−0.08±0.02	0.0006
ApoC-III, mg/dL	−0.17±0.02	<.0001	−0.15±0.03	<.0001
LDL size, nm	−0.01±0.001	<.0001	−0.01±0.002	<.0001
Lp(a), mg/dL	1.08±0.08	<.0001	1.08±0.10	<.0001
Lp-PLA_2_ mass, ng/mL	−0.18±0.02	<.0001	−0.20±0.03	<.0001
Lp-PLA_2_ activity, mol/min/mL	−0.09±0.02	<.0001	−0.14±0.02	<.0001
Leptin, ng/mL	0.28±0.03	<.0001	0.15±0.05	0.0014
Adiponectin, µg/mL	−0.29±0.03	<.0001	−0.34±0.04	<.0001
NT-proBNP, pg/mL	−0.45±0.04	<.0001	−0.29±0.05	<.0001
MR-proANP, pmol/L	−0.08±0.03	0.0120	−0.09±0.05	0.0513
CT-proAVP, pmol/L	0.47±0.04	<.0001	0.49±0.05	<.0001
MR-proADM, nmol/L	−0.03±0.02	0.2273	−0.08±0.02	0.0011
CT-proET, pmol/L	0.09±0.03	0.0017	0.10±0.03	0.0010
OPN, pg/mL	0.27±0.05	<.0001	0.12±0.06	0.0369
OPG, pg/mL	0.15±0.02	<.0001	0.15±0.03	<.0001
OCN, pg/mL	0.22±0.03	<.0001	0.07±0.04	0.1056
Factor II, % activity	0.06±0.01	<.0001	0.09±0.02	<.0001
Factor V, % activity	0.07±0.02	<.0001	0.08±0.02	0.0002
Factor VIII, % activity	0.12±0.02	<.0001	0.13±0.03	<.0001
vWF, IU/dL	0.18±0.03	<.0001	0.18±0.04	<.0001
D-dimer, ng/mL	0.18±0.04	<.0001	0.11±0.05	0.0440
Fibrinogen, mg/dL	0.15±0.02	<.0001	0.09±0.02	<.0001

Protein marker levels were natural log-transformed, stratified by sex and adjusted for age, BMI, smoking, hypertension, diabetes, myocardial infarction, stroke, total and HDL cholesterol, eGFR, systolic blood pressure, medication use (aspirin, estrogen, statin), alcohol, physical activity, education; no total and HDL cholesterol adjustment was done for ApoA-I, ApoB, ApoC-III, and ApoE.

As an internal check of validity, we assessed the ability of the protein markers to predict sex. We randomly split each cohort in half to create a training and test population within each group. The 47 markers predicted sex with 93% accuracy in both groups (c-statistic = 0.933 in AA and = 0.931 in NHW) with little degradation of performance between training and test samples. In addition, we repeated analyses with outliers removed rather than winsorized and found that our inferences did not change (analyses not shown).

## Discussion

It is becoming evident that for chronic, progressive diseases such as arteriosclerosis, multiple biomarkers will be needed to improve risk stratification, i.e. the “multimarker” approach. The value of using more than one marker has been illustrated in several studies. In the Atherosclerosis Risk in Communities study, patients in the highest tertile of Lp-PLA_2_ mass level were not associated with increased CV risk unless they also had elevated CRP levels [23]. In another study, the incidence of coronary heart disease was highest in the patients with both elevated CRP and D-dimer [24]. Zethelius et al [25]. found that 4 biomarkers from diverse etiologic pathways provided significantly improved prediction of future CV events compared to conventional risk factors in a cohort of elderly men. In the present study, we highlighted sex and ethnic differences in 47 candidate protein markers of CVD and our results may help direct future individualized risk-assessment and provide insights into pathophysiology.

### Sex Differences in Markers

After adjustment for potential confounders, levels of the acute phase reactants, CRP and SAA, adhesion molecule ICAM, pattern recognition receptor RAGE and the osteoclast-inhibiting cytokine OPG were higher in women, suggesting a pro-inflammatory state with upregulation of adhesion molecules [22]-[29]. There were also significant differences in the lipoprotein markers between the sexes. It is known that women and men have different baseline lipid profiles [26]. We found increased HDL-component ApoA-I in women along with the VLDL-component ApoC-III, Lp(a) and ApoE levels. There are known sex differences in baseline HDL cholesterol levels and recent evidence suggests lipoprotein markers have sex-specific relationships with subclinical vascular disease (e.g. intima-media thickness) [27]. An analysis from the Multi-Ethnic Study of Atherosclerosis study[28] compared post-menopausal women to men and found serum estradiol levels were associated with more atherogenic liproprotein profiles. Identifying differences between sexes in lipoprotein levels will help clarify their roles in health and disease.

Circulating levels of the adipokines leptin and resistin were higher in women than men, consistent with earlier reports [29], [30]. These adipokines are associated with truncal obesity and glucose intolerance and may contribute to increased activity of the nuclear factor kappa-light-chain-enhancer of activated B-cells (NFκB) pathway and subsequent cytokine production and upregulation of cell adhesion molecules [31], [32], [33], [34]. Consistent with previous reports, levels of the insulin-sensitizing and cardioprotective adipokine adiponectin were higher in women, possibly attenuating some of the deleterious effects of higher leptin levels [35], [36], [37]. Higher leptin levels have been reported to be more strongly associated with CVD in women than in men [38].

Significant sex differences were noted for plasma levels of the hemodynamic markers. CT-proAVP was significantly lower in women, possibly reflecting differences in hypothalamic downregulation of vasopressin by estrogen [39]. In contrast, NHW women had higher levels of the vasodilator natriuretic peptides NT-proBNP and MR-proANP and the vasodilator peptide, MR-proADM, suggestive of different baseline homeostatic set points that may have future implications for tailored antihypertensive pharmacotherapy. Higher natriuretic peptide levels have been associated with lower free testosterone levels[40] and there is significant extracardiac transcription of natriuretic peptides in the ovaries and uterus, even after menopause [41]. Another potential explanation is that women have lower plasma levels of renin, known to be inversely associated with natriuretic peptide levels [42].

Plasma levels of markers of calcification, OPG, OPN (in AA) and OCN (in AA) were higher in women than men. These factors are known to increase with bone remodeling and after “tissue” injury such as myocardial infarction [43], coronary artery disease[44] and osteoporosis[44]. We found higher OCN levels in AA women than in AA men, but lower levels in NHW women than NHW men. Previously, investigators have found lower plasma OCN levels in pre- vs. post-menopausal women[45] and lower OCN levels in premenopausal women compared to men[45], but higher OCN levels in postmenopausal women compared to men. These differences and the association of calcification markers with menopausal status point to a role of the endogenous sex hormones in influencing circulating OCN levels.

Factor II, V, and VII activities and levels of D-dimer and fibrinogen were higher in women than men. Among AA, women also had higher Factor VIII activity and increased levels of vWF compared to men. As Factor VIII is stabilized by vWF, this may reflect higher production or lesser degradation of vWF in AA women [46]. Higher levels of these biomarkers have been associated with increased CVD risk [47].

In summary, female sex was associated with higher levels of inflammatory markers, insulin-resistance promoting adipokines (leptin and resistin), natriuretic peptides, markers of calcification and coagulation factor levels and activity, potentially contributing to higher CVD risk (Table 6). While women traditionally have been considered to have overall lower CVD risk, in the postmenopausal setting, risk catches up with that of men [48]. It is unclear what contribution treatment differences vs. pathophysiologic differences make to this transition, but some of this “catch up” may be due to alterations in etiologic pathways that can be studied through circulating levels of protein markers. A recent study[49] from the Women's Health Initiative used 7 markers of inflammation and thrombosis to create a “Biomarker Risk Score” which improved risk-stratification for ischemic stroke, concluding that further investigation of multimarker panels was needed.

**Table 6:** Sex differences in women compared to men.

Both AA and NHW			AA			NHW		
Higher	Lower	No difference	Higher	Lower	No difference	Higher	Lower	No difference
CRP	TIMP-1	VCAM	RAGE		P-selectin	Hsp27	P-selectin	RAGE
SAA	Lp-PLA_2_ activity	IL-6	LDL size		Hsp27	MPO	Lp-PLA_2_ mass	LDL size
ICAM	CT-proAVP	IL-18	ONN		MPO	NT-proBNP	OCN	ONN
ApoA-I		TNFRI	OCN		Lp-PLA_2_ mass	MR-proANP		Factor VIII
ApoC-III		TNFRII	Factor VIII		NT-proBNP	CT-proET		vWF
ApoE		MCP-1	vWF		MR-proANP			
Lp(a)		E-selectin			CT-proET			
Leptin		MMP-2						
Adiponectin		MMP-9						
Resistin		TIMP-2						
MR-proADM		ApoB						
OPN		Ox-LDL						
Factor II		OPN						
Factor V								
Factor VII								
D-dimer								
ATIII								
Fibrinogen								

### Ethnic Differences

AA ethnicity was associated with higher levels of CRP and MPO, two inflammatory markers associated with higher CVD risk [50], [51], [52], [53], [54]. However, levels of several other markers in the NFκB pathway were lower in AA, including the cell adhesion molecules ICAM and VCAM, along with IL-18, TNFRI, and RAGE. These findings suggest ethnic differences in regulation of the NFkB pathway with potential diagnostic and therapeutic implications [55]. There was no significant association between AA ethnicity and levels of SAA, IL-6, E-selectin and TNFRII.

Differences in apolipoprotein levels between AA and NHW are well known and thought to be mediated partly by genetic polymorphisms [56], [57], [58]. In our study, AA subjects had favorable levels of ApoC-III and Lp-PLA_2_ mass and activity, but higher Lp(a) levels, highlighting differential hepatic processing and metabolism [59]. Higher Lp-PLA_2_ activity has been related to greater CVD risk[60] and lower Lp-PLA_2_ activity has been reported in AA [60].

Leptin levels were higher and adiponectin levels were lower in AA men and women than in their NHW counterparts, which may contribute to glucose intolerance and metabolic syndrome in AA individuals. Adiponectin levels are lower in AA youths than in their NHW counterparts[61] and have been associated with increased risk of diabetes even after adjustment for BMI, lifestyle factors, preexisting CVD, and systolic BP [62], providing a potential mechanism of increased susceptibility to diabetes and higher CVD risk in AA. In addition, it has been proposed that ethnic differences in the distribution of visceral vs. subcutaneous fat may be mediated by adiponectin and these differences may also contribute to increased CVD risk [63].

Among the hemodynamic markers, levels of the vasodilator, natriuretic peptide precursors NT-proBNP and MR-proANP were lower in AA whereas levels of the vasoconstrictive, antidiuretic peptides CT-proAVP and CT-proET peptides were higher. This may partially explain the clinical observations that AAs have a “salt retaining” phenotype and are more sensitive to vasopressin inhibition [64]. It is unclear whether the lower natriuretic peptide levels in AA in our study represent ethnic differences in the response to hemodynamic stress or a relative “deficiency” in baseline vasodilator natriuretic peptide levels. Our findings may help explain the higher propensity of hypertensive AA for adverse CVD events and may be useful in interpreting ethnicity-specific biomarker panels for CVD risk stratification.

Three of the four markers of calcification were higher in AA than in NHW participants. Emerging data suggest roles for OPN in atheroma formation, for OPG in vascular calcification, and for OCN in glucose homeostasis [65], [66]. We found higher levels of OCN in AA, in contrast with two previous reports suggesting lower OCN levels in AA [67], [68]. It is unclear how these markers of calcification may relate to lower prevalence of osteoporosis and coronary calcification in AA compared to NHW.

Markers of thrombosis were elevated in AA, including higher activities of Factors II, V, and VIII, and elevated levels of vWF, D-dimer and fibrinogen. These ethnic differences may increase propensity to thrombosis and contribute to higher CVD risk in AA [47]. The process and pathways involved in hemostasis and thrombosis are complex and have overlapping and intersecting limbs with the overall activity governed by the balance of activators and inhibitors.

In summary, AA ethnicity was associated with favorable levels of inflammatory markers and Ig superfamily adhesion molecules as well as several apolipoproteins and Lp-PLA_2_ mass and activity. AA ethnicity was associated with potentially unfavorable levels of 23 markers, including inflammatory biomarkers, adipokines (leptin and adiponectin), hemodynamic markers and markers of calcification and thrombosis, including D-dimer and fibrinogen (Table 7). While AA and NHW subjects were recruited from geographically distinct locations and differed significantly in baseline characteristics, we adjusted for both conventional and lifestyle factors to minimize the impact of these covariates. The differences we have identified in levels of candidate protein markers may represent true ethnic differences in physiology and pathophysiology and motivate further investigation.

**Table 7:** African American ethnicity differences in AA compared to NHW.

Both women and men			Women			Men		
Increased	Decreased	No difference	Increased	Decreased	No difference	Increased	Decreased	No difference
CRP	ICAM	SAA	MCP-1	MR-proANP	MR-proADM		MR-proADM	MCP-1
Hsp27	VCAM	IL-6	P-selectin					P-selectin
MPO	IL-18	TNFRII	MMP-2					MMP-2
Lp(a)	TNFRI	E-selectin	TIMP-2					TIMP-2
Leptin	RAGE	ApoA-I	OCN					MR-proANP
CT-proAVP	MMP-9	ApoE						OCN
CT-proET	TIMP-1	Resistin						
OPN	ApoB	ONN						
OPG	ApoC-III	Factor VII						
Factor II	LDL size	ATIII						
Factor V	Lp-PLA_2_ mass							
Factor VIII	Lp-PLA_2_ activity							
vWF	Adiponectin							
D-dimer	NT-proBNP							
Fibrinogen								

### Study Limitations

Although our study included a relatively large sample size, multiple candidate markers, quality control measures of intra-assay imprecision, adjustment for conventional and lifestyle factors and the inclusion of two ethnic groups, our results will require further validation before being applied in clinical practice. In addition, several limitations need to be acknowledged. First, although we measured multiple markers representative of etiologic pathways implicated in arteriosclerotic vascular disease, other unmeasured markers may be important in influencing risk of disease. Second, the analytical precision of the assays varied, and this may have influenced our results. In general, assay imprecision reflected the robustness of the measurement technology and we excluded data based on a quality control program as previously detailed [13]. Multiplicity of testing with 47 biomarkers in an individual sample also has unique statistical challenges in the imputation of missing data and interpretation of analyses. Third, our analysis is cross-sectional, with markers measured at only one point in time and does not imply directionality in the associations between markers and sex and ethnicity, notwithstanding the biological plausibility of such associations. It is also possible that activation of these pathways may be a consequence of arteriosclerosis (“reverse” causality) and that potentially unknown confounders and contributors were not adjusted for. AA and NHW populations were recruited from differing geographical locations and it is possible that unmeasured environmental and geographic factors contributed to purported ethnic differences. There are sparse data in the literature regarding geographic differences in biomarker levels, although environmental differences linked to pollution exposure appear to influence circulating levels of inflammatory markers [69], [70]. Finally, the associations described in this study may not be generalizable to younger and normotensive adults.

### Conclusions

We found significant sex and ethnic differences in circulating levels of multiple novel candidate protein markers of CVD independent of potential confounding variables. We performed these analyses as a step towards identifying clinically useful panels of for refining CVD risk assessment. The results will help guide subsequent investigation of the association of the markers individually and by pathway with objective measures of subclinical vascular disease as well as with adverse cardiovascular events.

## Supporting Information

Table S1 Biomarkers, Method, and Precision of Assay.
(0.13 MB DOC)
