# Supplementary material for: Sex and Ethnic Differences in 47 Candidate Proteomic Markers of Cardiovascular Disease: The Mayo Clinic Proteomic Markers of Arteriosclerosis Study
Source: PLoS One. 2010 Feb 5;5(2):e9065. doi: 10.1371/journal.pone.0009065 (PMC2816715; doi:10.1371/journal.pone.0009065)
Supplement: Table S1 — Biomarkers, Method, and Precision of Assay. (0.13 MB DOC) [file pone.0009065.s001.doc]

| **Table S1. Biomarkers, Method, and Precision of Assay.** | | | | |
| --- | --- | --- | --- | --- |
| Analyte | Method | Inter-assay imprecision  CV %, (levels) | Intra-assay imprecision  CV%, (levels) | Sample matrix |
| **Inflammation** | | | | |
| CRP, mg/L | Immunoturbidometric^1^ | 1.8-2.6%  (3.9-10.9 mg/L) | 1.0-9.2%  (0.56-5.2 mg/L) | Serum |
| SAA, µg/mL | ELISA^2^ | 9-14%  (15.3-406 µg/mL) | 3-11.5%  (31.3-384 µg/mL) | EDTA plasma |
| ICAM, ng/mL | ELISA^3^ | 11.4-14.1%  (284-645 ng/mL) | 3.6-4.3%  (324-590 ng/mL) | EDTA plasma |
| VCAM, ng/mL | ELISA^3^ | 5.3-9.8%  (62.6-868 ng/mL) | 4.5-7.3%  (183-437 ng/mL) | EDTA plasma |
| IL-6, pg/mL | ELISA 6-plex^4^ | 25.6%  (4.1 pg/mL) | 12.4%  (4.6 pg/mL) | EDTA plasma |
| IL-18, pg/mL | ELISA 6-plex^4^ | 29.9%  (35.5 pg/mL) | 14.3%  (35.7 pg/mL) | EDTA plasma |
| TNFRI, pg/mL | ELISA 6-plex^4^ | 29.2%  (818 pg/mL) | 9.4%  (971.9 pg/mL) | EDTA plasma |
| TNFRII, pg/mL | ELISA 9-plex^4^ | 27.4%  (1,191 pg/mL) | 10.9%  (1,200 pg/mL) | EDTA plasma |
| MCP-1, pg/mL | ELISA 9-plex^4^ | 32.4%  (623 pg/mL) | 13.1%  (745 pg/mL) | EDTA plasma |
| E-selectin, ng/mL | ELISA 9-plex^4^ | 18.2%  (58.8 ng/mL) | 8.3%  (60.4 ng/mL) | EDTA plasma |
| P-selectin, ng/mL | ELISA 6-plex^4^ | 17.9%  (39.7 ng/mL) | 7.1%  (40.4 ng/mL) | EDTA plasma |
| Hsp27, ng/mL | ELISA^5^ | 4.1-12.3%  (491-1578 ng/mL) | 11.2-12.6%  (1594-3783 ng/mL) | Serum |
| MPO, ng/mL | ELISA^6^ | 7.2-7.4%,  (4.1-166 ng/mL) | 10.7-12.4%  (2.8-52.2 ng/mL) | Serum |
| RAGE, pg/mL | ELISA 6-plex^4^ | 30.1%  (1,021 pg/mL) | 14.5%  (1,038 pg/mL) | EDTA plasma |
| MMP-2, ng/mL | ELISA 9-plex^4^ | 26.9%  (1,359 ng/mL) | 9.5%  (1,398 ng/mL) | EDTA plasma |
| MMP-9, ng/mL | ELISA 9-plex^4^ | 22.6%  (155.5 ng/mL) | 54.3%  (119.2 ng/mL) | EDTA plasma |
| TIMP-1, ng/mL | ELISA 9-plex^4^ | 20%  (62.4 ng/mL) | 5.4%  (61.7 ng/mL) | EDTA plasma |
| TIMP-2, ng/mL | ELISA 9-plex^4^ | 25.8%  (121.7 ng/mL) | 8%  (121.9 ng/mL) | EDTA plasma |
| **Lipoprotein metabolism** | | | | |
| ApoA-I, mg/dL | Immunoturbidometric^1^ | 5-6%  (95-259 mg/dL) | 0.7-1.0%  (90-230 mg/dL) | EDTA plasma |
| ApoB, mg/dL | Immunoturbidometric^1^ | 3-6%  (45-163 mg/dL) | 1-2.3%  (65-144 mg/dL) | EDTA plasma |
| ApoC-III, mg/dL | Immunoturbidometric^1^ | 8.1-8.4%  (8.5-23 mg/dL) | 0.9-1.5%  (9.3-24.6mg/dL) | EDTA plasma |
| ApoE, mg/dL | Immunoturbidometric^1^ | 10-12%  (1.3-3.5 mg/dL) | 1.9-2.9%  (2.7-3.5 mg/dL) | EDTA plasma |
| LDL size, nm | Electrophoresis^7^ | 0.47-0.79%  (25.6-26.5 nm) | x | EDTA Plasma |
| Lp(a), mg/dL | Immunoturbidometric^1^ | 6.5-6.7%  (22.5-53.5 mg/dL) | 1.2-2.1%  (19-45 mg/dL) | Serum |
| Ox-LDL, U/L | ELISA^8^ | 4.8-8.4%  (3.8-10.9 U/L) | 4.4-7.1%  (2.0-2.5 U/L) | EDTA Plasma |
| Lp-PLA_2_ mass, ng/mL | ELISA^9^ | 5.3-10.3%  (84.4-230.1 ng/mL), | 10.5-15.1%  (190-402 ng/mL) | Serum |
| Lp-PLA_2_ activity, mol/min/mL | Chromogenic^9^ | 5.7-8.3%  (98.8-223 mol/min/mL) | x | Serum |
| **Adipocyte metabolism** | | | | |
| Leptin, ng/mL | RIA^10^ | 11-13%  (3.0-20.4 ng/mL) | 6.1-7.7%  (3.8-39.7 ng/mL) | EDTA Plasma |
| Adiponectin, µg/mL | RIA^10^ | 13.0-18.4%  (1.6-11.6 µg/mL) | 4.7-17.0%  (5.1-119 µg/mL) | EDTA Plasma |
| Resistin, ng/mL | ELISA^11^ | 11.8-12.9%  (6.6-17.5 ng/mL) | 6.7-10.3%  (3.0-15.4 ng/mL) | EDTA Plasma |
| **Hemodynamics** | | | | |
| NT-proBNP, pg/mL | ELISA^4^ | 42.1%  (87.4 pg/mL) | 9.1%  (80 pg/mL) | EDTA plasma |
| MR-proANP, pmol/L | Immunoluminometric^12^ | < 20%  (18-65 pmol/L) | <10%  (23-3000 pmol/L) | EDTA plasma |
| CT-proAVP, pmol/L | Immunoluminometric^12^ | <15%  (2.25-420 pmol/ L) | <10%  (2.25-420 pmol/ L) | EDTA plasma |
| MR-proADM, nmol/L | Immunoluminometric^12^ | < 10%  (0.14-14.1 nmol/L) | < 7%  (0.08-14.7 nmol/L) | EDTA plasma |
| CT-proET, pmol/L | Immunoluminometric^12^ | < 10%  11.6-317 pmol/L | <5%  11.7-206 pmol/L | EDTA plasma |
| **Calcification** | | | | |
| OPN, pg/mL | Immunoassay triplex^13^ | 24.9%  (11.9 pg/mL) | 10.8-11.4%  (10.1-13.5 pg/mL) | Serum |
| OPG, pg/mL | Immunoassay^13^ | 2.5-4.0%  (133-180 pg/mL) | 11.3-13.3%  (149-189 pg/mL) | Serum |
| ONN, pg/mL | Immunoassay triplex^13^ | 15.9-18.6%  (864-937 pg/mL) | 5.3-6.3%  (672-926 pg/mL) | Serum |
| OCN, pg/mL | Immunoassay triplex^13^ | 5.8-21.1%  (13.4-32.5 pg/mL) | 5.7-5.8%  (12.7-32.5 pg/mL) | Serum |
| **Thrombosis** | | | | |
| Factor II, % activity | Clot-based^14^ | 6.4-8%  (41.6-98.6%) | 4.1-4.4%  (35.8-81.3%) | Sodium-citrate plasma |
| Factor V, % activity | Clot-based^14^ | 4.9-11%  (41.3-111%) | 4.7-6.5%  (47.5-113) | Sodium-citrate plasma |
| Factor VII, % activity | Clot-based^14^ | 10-11%  (41.3-101.6%) | 4.1-4.4%  (35.8-81.3%) | Sodium-citrate plasma |
| Factor VIII, % activity | Clot-based^14^ | 17.9-18%  (43.3-105%) | 6.3-6.5%  (47.5-113%) | Sodium-citrate plasma |
| vWF, IU/dL | ELISA^14^ | 2.9- 9.2%  (10.9-162%) | 4.5-9.5%  (34-70.3%) | Sodium-citrate plasma |
| D-dimer, ng/mL | Immunoturbidometric^14^ | 4-15%  (244-2061 ng/mL) | 3.1-24.4%  (100-1071 ng/mL) | Sodium-citrate plasma |
| ATIII, % activity | Chromogenic^14^ | 7.6-7.8%  (45.2 -104 %) | 3.8-3.9%  (45.5-105%) | Sodium-citrate plasma |
| Fibrinogen, mg/dL | Clot-based^14^ | 2.5-3.5%  (111-286 mg/dL) | 2.4-5.3%  (110-282 mg/dL) | Sodium-citrate plasma |
| 1. Diasorin, Inc., Stillwater, MN; 2. BioSource International, Camarillo, CA; 3. R&D Systems, Minneapolis, MN, 4. SearchLight™, Pierce, Boston, MA; 5. Calbiochem, Gibbstown, NJ; 6. ALPCO Diagnostics, Salem, NH; 7. Lipoprint™ LDL System; Quantimetrix, Redondo Beach, CA; 8. Mercodia, Uppsala, Sweden; 9. Diadexus, South San Francisco, CA; 10. Linco Research, Inc, St. Louis, MO; 11. BioVendor, Modrice, Czech Republic; 12. BRAHMS, Hennigsdorf/Berlin, Germany; 13. Meso-scale Discoveries, Gaithersburg, MD; 14. Diagnostica STAGO, Asnieres, France | | | | |
